# C-852-08. An Analysis of Hypermetabolic Response in Patients with Less Than 20% TBSA Burn Injuries

**DOI:** 10.1093/jbcr/irag033.068

**Published:** 2026-04-06

**Authors:** Stacey M Knowles, Alexis Kimball, Anastasiya Ivanko, M Victoria P Miles, Jeffrey E Carter, Jonathan E Schoen, Aaron Hong

**Affiliations:** University Medical Center Burn Center, Louisiana State University Health Sciences Center, New Orleans, LA; University Medical Center Burn Center, Louisiana State University Health Sciences Center, New Orleans, LA; University Medical Center Burn Center, Louisiana State University Health Sciences Center, New Orleans, LA; University Medical Center New Orleans, Louisiana State University Health Sciences Center, New Orleans, LA; University Medical Center Burn Center, Louisiana State University Health Sciences Center, New Orleans, LA; UCI Health Regional Burn Center, New Orleans, LA; University Medical Center Burn Center, Louisiana State University Health Sciences Center, New Orleans, LA

## Abstract

**Introduction:**

Burn injuries involving ≥20% total body surface area (TBSA) are classically associated with a hypermetabolic response, characterized by elevated resting energy expenditure (REE), catabolism, and immune dysregulation. However, the extent to which moderate burns (10–19% TBSA) induce hypermetabolism remains unclear. This study aimed to evaluate the presence and degree of hypermetabolic response in patients with moderate burn injuries.

**Methods:**

A prospective analysis was conducted on burn patients (10-19% Total Burn Surface Area (TBSA)) from July 2024 to July 2025 at a single, ABA-verified burn center. Indirect calorimetry was performed at weekly intervals while inpatient and monthly intervals while outpatient to measure REE and hypermetabolism. Measured REE values were compared with predicted REE calculated using standard predictive equations. The degree of hypermetabolism was expressed as greater than +10% increase over predicted REE of a healthy individual with the same age, height, and weight.

**Results:**

A total of 19 patients met inclusion criteria for the study period. Overall, patients with 10–19% TBSA burns demonstrated significant elevations in measured REE compared with predicted values. Out of the 19 patients who received indirect calorimetry testing, only 3 of these patients did not experience hypermetabolism, two of these being women. Propranolol was initiated in 63% (n = 12) of the patients evaluated. On average, REE was 34% higher than normal, with 75% of patients showing a sustained hypermetabolic state throughout hospitalization. This hypermetabolic response persisted for up to 3.5 weeks post-injury, a pattern similar to that observed in larger burns (≥ 20% TBSA).

**Conclusions:**

These findings demonstrate that hypermetabolism may not be restricted to burns ≥20% TBSA. Patients with moderate burns (10–19% TBSA) may exhibit a profound and sustained hypermetabolic response. Early recognition of this metabolic demand is essential for guiding nutritional interventions and optimizing recovery strategies in this patient population. Additional research is warranted to correlate hypermetabolic markers in this patient population with attention to different age cohorts.

**Applicability of Research to Practice:**

This research underscores the importance of reevaluating the clinical approach to burn injuries, particularly for patients with moderate burns (< 20% TBSA). By recognizing the hypermetabolic response early, adjusting nutritional strategies, and tailoring recovery plans, healthcare providers can improve patient outcomes.

**Funding for the study:**

N/A.

